# Early neonatal diagnosis of SSR4-related congenital disorder of glycosylation with severe congenital heart defects: a case report and systematic review

**DOI:** 10.3389/fped.2026.1780997

**Published:** 2026-03-25

**Authors:** Lingxia Zhao, Lingkong Zeng, Minghui Yi, Wenhao Yuan

**Affiliations:** 1 Department of Neonatology, Wuhan Children’s Hospital (Wuhan Maternal and Child Healthcare Hospital), Tongji Medical College, Huazhong University of Science and Technology, Wuhan, Hubei, China; 2 Department of Radiology, Wuhan Children’s Hospital (Wuhan Maternal and Child Healthcare Hospital), Tongji Medical College, Huazhong University of Science and Technology, Wuhan, Hubei, China

**Keywords:** CDG1Y, congenital disorders of glycosylation, congenital heart defects, contiguous gene deletion, neonatal diagnosis, SSR4-CDG, TRAP complex, x-linked disorder

## Abstract

**Background:**

Congenital disorders of glycosylation type Iy (SSR4-CDG, CDG1Y) is an ultra-rare X-linked disorder caused by pathogenic variants in the *SSR4* gene, encoding a subunit of the translocon-associated protein (TRAP) complex. While typically recognized for neurodevelopmental and facial features, its full neonatal spectrum, particularly regarding cardiac involvement, remains under-characterized.

**Case presentation:**

We report a male neonate with the earliest postnatal diagnosis of SSR4-CDG (day of life 6). Prenatal testing revealed a novel, maternally inherited 65.63 kb hemizygous deletion at Xq28, encompassing *SSR4* and partially deleting *ABCD1*. The neonatal presentation was dominated by multiple congenital heart defects (CHDs): Membranous ventricular septal defect, secundum atrial septal defect, persistent left superior vena cava, a narrow proximal left pulmonary artery, and coronary sinus dilation. Additional features included classic dysmorphism (wide mouth, deep-set eyes, micrognathia), severe hypotonia, feeding difficulties, and coagulopathy. Brain MRI revealed a thin corpus callosum.

**Literature review & analysis:**

A systematic review of the literature, including reports published up to December 2025, identified 24 previously published cases. Pooled analysis incorporating the present patient (*n* = 28) confirmed that developmental delay/intellectual disability, hypotonia, characteristic facial features, and microcephaly were observed in 100% of cases. Congenital heart defects (CHDs) were present in 32.1% (9/28) of patients; however, the current case represents the first reported patient in whom severe CHDs constituted the predominant clinical manifestation. Detailed subgroup analyses further demonstrated that the frequency of clinical features varied across different age groups, indicating age-dependent phenotypic expression. All analyses were descriptive in nature, and no formal meta-analysis was performed due to the limited number of reported cases and heterogeneity in clinical data.

**Conclusions:**

This case expands the neonatal phenotype of SSR4-CDG and highlights that, in some patients, severe congenital heart defects may represent an early and clinically significant manifestation. However, based on currently available evidence, cardiac anomalies remain an uncommon feature of the disorder. Prompt genetic evaluation should be considered in affected male neonates with syndromic features.

## 1 Introduction

Congenital disorders of glycosylation (CDG) represent a vast, genetically heterogeneous group of over 180 inherited metabolic diseases resulting from defects in the intricate pathways responsible for the synthesis, processing, and attachment of glycans to proteins and lipids (1–3). As glycans are fundamental for protein folding, stability, trafficking, and function, CDG manifests as complex multi-system disorders with a pronounced neurological burden, though virtually any organ can be affected (4, 5). The diagnostic landscape has been revolutionized by next-generation sequencing, yet phenotypic recognition remains crucial for guiding targeted testing.

CDG type Iy (CDG1Y, OMIM 300934), also termed SSR4-CDG, is an ultra-rare, X-linked recessive subtype. It is caused by loss-of-function variants in the *SSR4* gene (Signal Sequence Receptor 4, OMIM 300090) located at Xq28 (6). Since its inaugural description in 2014 (7), only 27 cases have been documented in the literature to date, with the present case bringing the total number of reported patients to 28 (6–12). The established clinical portrait is that of a neurodevelopmental disorder: affected males exhibit global developmental delay, intellectual disability, muscular hypotonia, progressive microcephaly, and a distinctive facial gestalt comprising deep-set eyes, a wide mouth with a thin upper lip, large ears, and micrognathia (13, 14). Non-neurological features include feeding difficulties, failure to thrive, ophthalmological issues (strabismus), connective tissue abnormalities (joint laxity, redundant skin), skeletal anomalies, and behavioral problems (13, 15). Cardiac anomalies, while reported sporadically, have not been emphasized as a cardinal or presenting feature.

Here, we present an early neonatal case of SSR4-related congenital disorder of glycosylation (SSR4-CDG) diagnosed on the sixth day of life, representing the earliest postnatal diagnosis reported to date. Uniquely, the clinical presentation was characterized by early and clinically significant congenital heart defects (CHDs),for which elective, staged medical and/or surgical intervention was recommended. Furthermore, the underlying genetic abnormality was a novel, large contiguous gene deletion involving SSR4 and partially affecting the adjacent ABCD1 gene, which is implicated in X-linked adrenoleukodystrophy (ALD). This case prompts a re-evaluation of the neonatal phenotypic spectrum of SSR4-CDG. In addition, we complement this report with a comprehensive and up-to-date systematic review of all published SSR4-CDG cases, providing an age-stratified synthesis of clinical manifestations and highlighting patterns of phenotypic evolution across the lifespan. We therefore report an early neonatal case of SSR4-CDG with severe congenital heart disease and provide a systematic review to better define the age-dependent clinical spectrum.

## 2 Pathophysiology: the TRAP complex, N-glycosylation, and consequences of SSR4 deficiency

To appreciate the multisystem pathology of SSR4-CDG, a understanding of the underlying molecular defect is essential. Protein N-glycosylation is a critical co- and post-translational modification occurring in the endoplasmic reticulum (ER). The process begins with the synthesis of a lipid-linked oligosaccharide (LLO) precursor, which is then *en bloc* transferred to specific asparagine residues (Asn-X-Ser/Thr sequon) of nascent polypeptide chains entering the ER lumen. This transfer is catalyzed by the oligosaccharyltransferase (OST) complex (16).

The translocation of nascent chains across the ER membrane is facilitated by the Sec61 translocon. The translocon-associated protein (TRAP) complex, a heterotetramer composed of SSR1, SSR2, SSR3, and SSR4 (also known as TRAP-α, -β, -γ, and -δ, respectively), is intimately associated with the Sec61 channel and the OST complex (17, 18). Cryo-electron microscopy studies reveal that the TRAP complex, particularly via its SSR4 subunit, sits at the interface between the translocon and OST, acting as a structural scaffold and a regulatory module (19, 20). It is thought to enhance the efficiency and fidelity of protein translocation and directly facilitate the N-glycosylation process by optimally positioning the OST complex relative to the translocating peptide chain.

*SSR4* encodes a 173-amino acid transmembrane protein essential for the stability and function of the entire TRAP complex. Loss-of-function variants in *SSR4* lead to destabilization of the complex, as demonstrated by reduced levels of other SSR subunits in patient-derived fibroblasts (7, 21). This destabilization impairs the co-translational glycosylation machinery. Consequently, a broad spectrum of glycoproteins fails to acquire their proper glycan chains, leading to protein misfolding, aberrant trafficking, accelerated degradation, and ultimately, loss of function (22). The biochemical hallmark is a “Type I” pattern on serum transferrin isoelectric focusing (TIEF) or carbohydrate-deficient transferrin (CDT) analysis, reflecting the loss of entire glycan moieties due to defects in the early ER steps of glycosylation.

The multisystem manifestations of SSR4-CDG arise from this global glycoprotein deficiency. Neurological symptoms likely stem from impaired glycosylation of crucial receptors, adhesion molecules, and channels in the developing brain. Connective tissue abnormalities may result from defects in the glycosylation of extracellular matrix components like collagens and fibrillins. The cardiac defects observed in our patient and others suggest that key morphogens, signaling receptors (e.g., Notch, Fibroblast Growth Factor receptors), or structural proteins essential for heart development are sensitive to the glycosylation deficit imposed by *SSR4* deficiency. Similarly, coagulation factors are heavily glycosylated, explaining the reported coagulopathies in a subset of patients.

## 3 Case presentation

### 3.1 Prenatal history and genetic findings

The proband was the first child of a non-consanguineous Chinese couple (father: 30y, mother: 25y). The pregnancy was conceived naturally. Due to a non-specific family history of learning difficulties and advanced maternal age, an amniocentesis with chromosomal microarray analysis (CMA) was performed at 32 weeks' gestation. CMA revealed a novel, maternally inherited, 65.63 kb hemizygous deletion at chromosome Xq28: arr[GRCh38] Xq28(153,739,978_153,805,606)x1. This deletion was predicted to fully encompass the *SSR4* gene (all exons) and partially delete the 3’ end of the adjacent *ABCD1* gene (exons 6–10) along with five neighboring genes, including PLXNB3, SRPK3, IDH3G, and PDZD4. Comprehensive genetic counseling detailed the X-linked inheritance, the high likelihood of a severe phenotype in a male fetus, the variable expressivity in female carriers, and the potential implications of the *ABCD1* partial deletion. After deliberation, the parents opted to continue the pregnancy.

### 3.2 Perinatal course and initial presentation

The infant was delivered via elective cesarean section at 38 weeks and 4 days due to breech presentation. Apgar scores were 6 at 1 min and 8 at 5 min. Birth parameters indicated growth restriction: weight 2.26 kg (<10th percentile), length 45 cm (<10th percentile), head circumference 31 cm (<3rd percentile). He required brief non-invasive respiratory support for transient distress. A routine neonatal echocardiogram on day 2 revealed complex CHDs: a 8 mm perimembranous ventricular septal defect (VSD) with left-to-right shunting, a 5 mm secundum atrial septal defect (ASD), and a persistent left superior vena cava (PLSVC) draining into the coronary sinus, Dilation of the coronary sinus [width: [4.5] mm/m^2^, normal <[3.5] mm/m^2^(body surface area-adjusted)]. Mild narrowing of the aortic isthmus [diameter: [3.5] mm, reference range: [4–7] mm] Hypoplasia of the left pulmonary artery [LPA diameter: [2.5] mm, reference range: [3.95–5.95] mm]. The infant was transferred to our hospital on the 6th day of life due to persistent tachypnea at rest and perioral cyanosis during crying. After admission, treatment included digoxin for cardiac support, formula feeding with gradual volume advancement, intravenous fluid supplementation, anti-infective therapy, and non-invasive high-flow nasal cannula oxygen (stepped down to standard nasal cannula after 3 days). Following 10 days of comprehensive management, the infant's condition improved, and he was discharged.

Based on the prenatal amniocentesis CMA findings and the current clinical presentation, the infant was highly suspected of having Congenital Disorder of Glycosylation type 1y (SSR4-CDG). After thorough communication and obtaining informed consent from the parents, whole-exome sequencing (trio analysis) was performed on peripheral blood samples from the infant and both parents. A multidisciplinary team consultation was also recommended to establish a standardized management plan. The parents did not accept the preliminary diagnosis at that time and declined systematic case data collection and facial photography. Once the infant's respiratory status stabilized, the parents signed for discharge. Due to continued poor weight gain after discharge, the infant was readmitted at 35 days of age for further evaluation and intervention.

### 3.3 Clinical examination on admission

**Figure 1:**
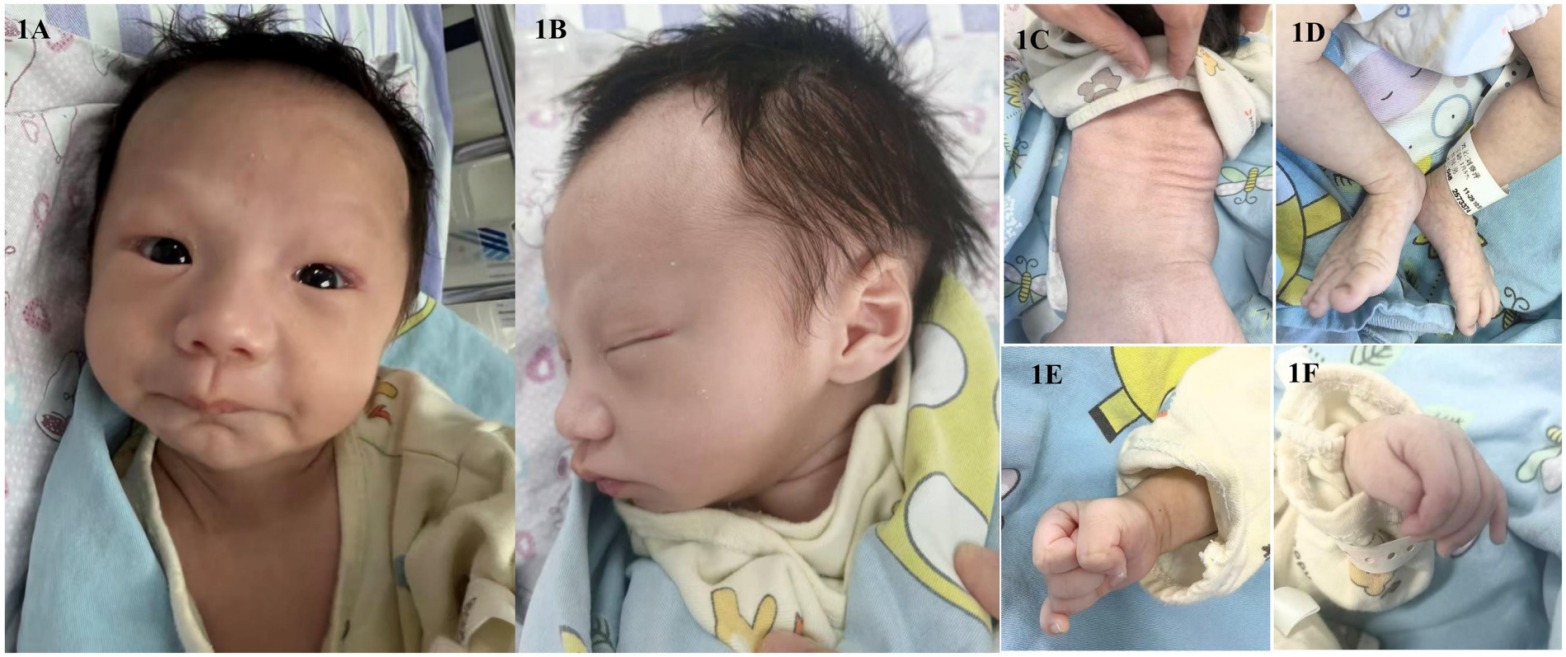
Clinical photographs of a neonate with SSR4-CDG (neonatal-onset), showing the face, trunk, and extremities. **(A,B)** (Frontal and lateral facial views): The examination revealed a disproportionately large and wide mouth with thin vermilion borders, deep-set eyes, mild hypertelorism, a hypoplastic and retracted jaw (microretrognathia), a low and broad nasal bridge, and a prominent forehead. The auricles were normally shaped and positioned. **(C–F)** No cutaneous abnormalities, such as abnormal dorsal fat pads, were observed. There was no evidence of scoliosis, clinodactyly, or talipes valgus deformity.

Examination on admission [weight 2.44 kg(<3rd percentile), head circumference 34.5 cm (4th percentile), and length 51 cm (5th percentile).] revealed a critically ill neonate with striking dysmorphic features (Figure 1): a disproportionately wide mouth (macrostomia) with a thin upper vermilion border, deep-set eyes, mild hypertelorism, a prominent forehead, a low and broad nasal bridge, and severe micrognathia with retrognathia. The ears were large but normally formed. He exhibited profound generalized hypotonia with a weak suck and absent Moro reflex. Cardiac auscultation confirmed a loud grade 3/6 holosystolic murmur. There were no contractures, spinal deformities, or abnormal skin folds.

### 3.4 Diagnostic investigations

#### 3.4.1 Laboratory studies

Coagulation screening revealed a significant coagulopathy: antithrombin III activity was low at 55.6% (ref 70%–130%), fibrinogen was decreased at 1.08 g/L (ref 1.92–4.01 g/L), and activated partial thromboplastin time (APTT) was prolonged at 40.2 s (ref 21.1–36.5). Complete blood count, electrolytes, renal/liver function, ammonia, lactate, thyroid function, and newborn metabolic screening (tandem mass spectrometry for amino acids/acylcarnitines and urine organic acids) were unremarkable.Serum transferrin isoelectric focusing (TIEF) or carbohydrate-deficient transferrin (CDT) analysis was not performed in this patient. The diagnosis was established based on prenatal chromosomal microarray findings and confirmed by trio-based genomic sequencing demonstrating complete SSR4 deletion, which represents a known loss-of-function mechanism for SSR4-CDG. Given the definitive molecular diagnosis, additional biochemical confirmation was not pursued.

#### 3.4.2 Imaging and functional studies

**Figure 2:**
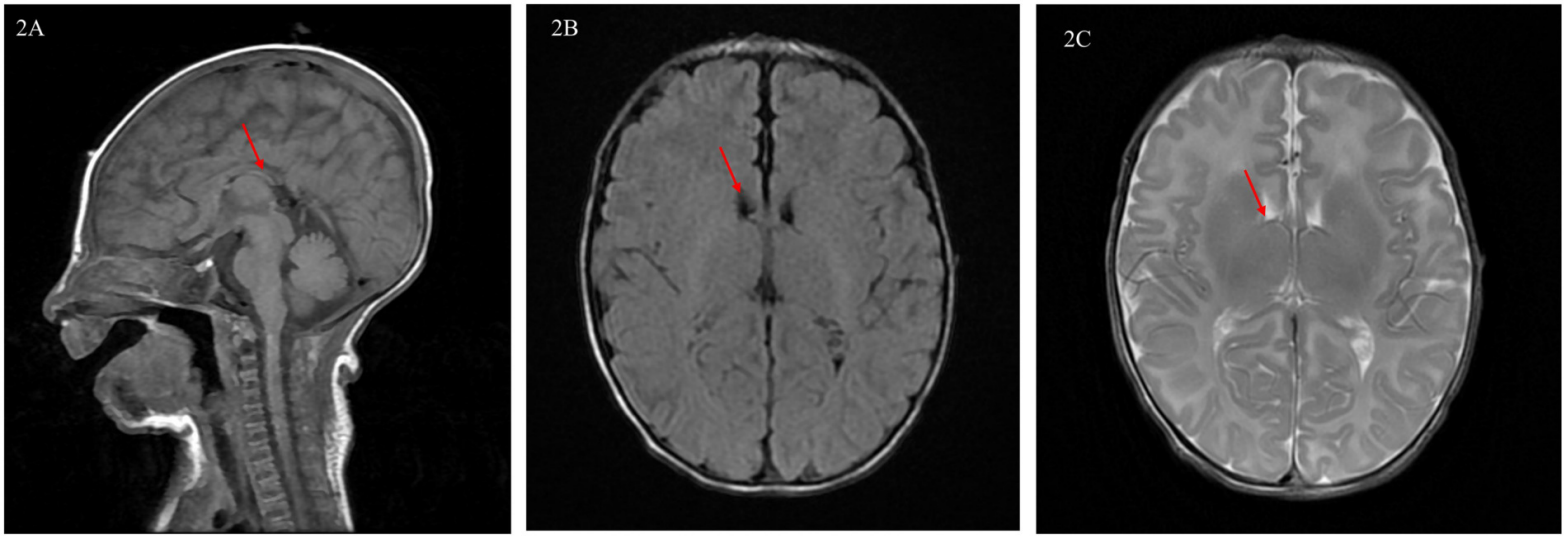
Brain MRI of a newborn with SSR4-CDG (onset in the neonatal period). **(A)** T1-weighted sagittal images suggest: Short corpus callosum (GE-3.0T). **(B)** T2-FLAIR axial images demonstrate mild contour irregularity of the bilateral lateral ventricles. **(C)** Axial T2-weighted images demonstrate mild irregularity in the contour of both lateral ventricles (as indicated by the arrows).

- **Cardiac Imaging:** Transthoracic echocardiography revealed an 8 mm perimembranous ventricular septal defect with left-to-right shunting, a 5 mm secundum atrial septal defect, persistent left superior vena cava draining into the coronary sinus with associated dilation, and mild narrowing of the proximal left pulmonary artery. Cardiac computed tomography angiography (CTA) confirmed these findings and provided detailed anatomical delineation, with defect dimensions concordant with echocardiographic measurements (Supplementary Figure S1).
- **Cranial MRI:** Demonstrated a thin corpus callosum, most notably in the posterior body and splenium, and mild irregularity of the lateral ventricular contours (Figure 2). Myelination was appropriate for gestational age.
- **Video EEG:** Background activity was appropriate for conceptional age with no epileptiform discharges.
- **Abdominal Ultrasound:** Normal.
- **Brainstem Auditory Evoked Potentials (BAEP):** Showed prolonged wave III and V latencies bilaterally, suggesting brainstem conduction abnormality.

#### 3.4.3 Genetic confirmation

**Figure 3:**
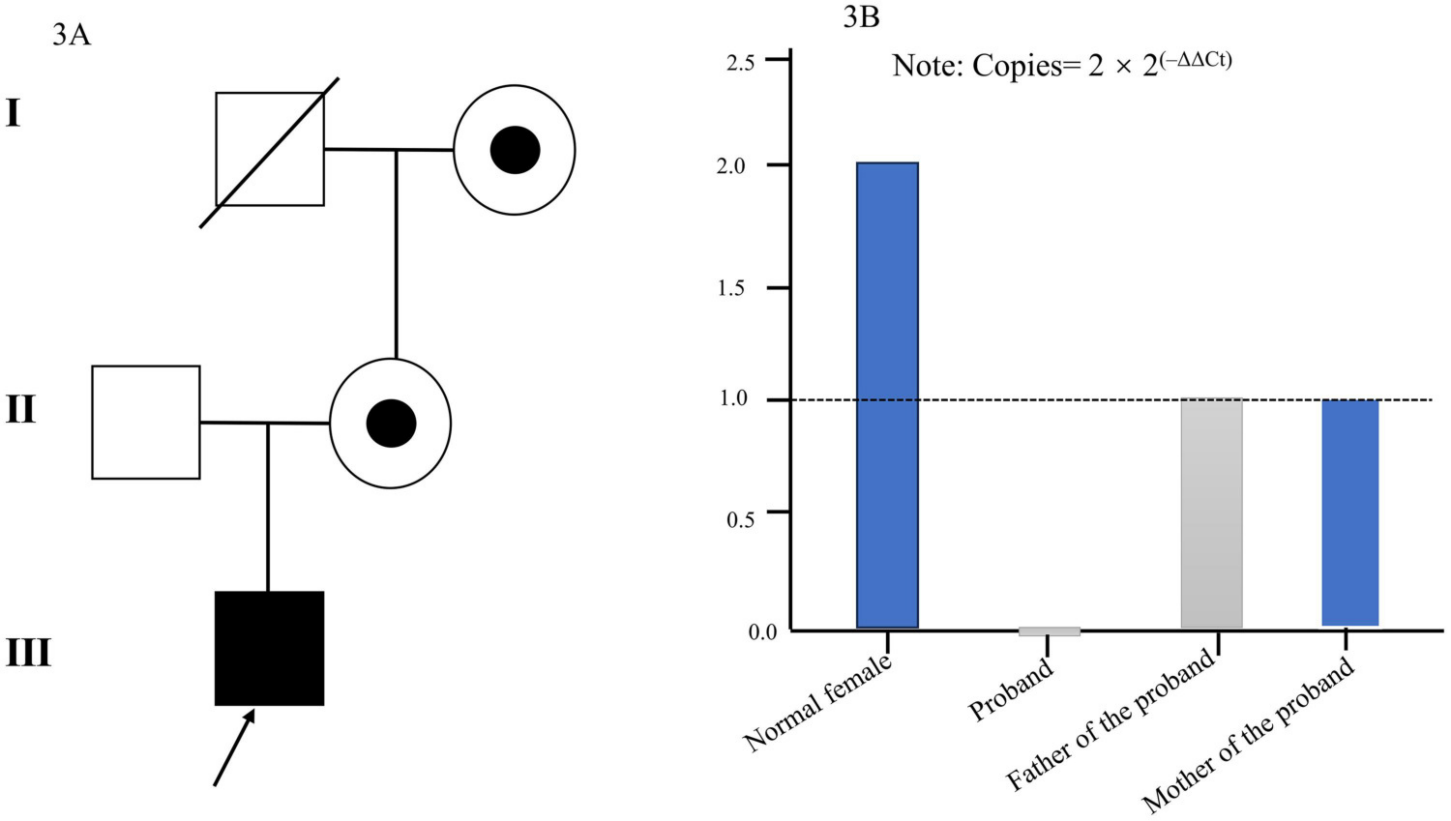
Pedigree and copy number variation analysis reveal a *de novo* deletion in a patient with SSR4-CDG. **(A)** Pedigree of the family. The arrow indicates the proband (affected male). **(B)** Validation of a hemizygous deletion on chromosome X.

Trio-based whole-genome sequencing with CNV analysis corroborated the prenatal findings. The proband's hemizygous deletion and the mother's heterozygous status were validated by quantitative PCR (qPCR) (Figure 3). The father's genotype was normal. Following ACMG/ClinGen guidelines, the CNV was classified as **Pathogenic** (PVS1, PS3_moderate, PM2_Supporting). The *SSR4* deletion is a known haploinsufficient mechanism for SSR4-CDG. The partial *ABCD1* deletion is also considered pathogenic for ALD, given the gene's haploinsufficiency score of 3 in ClinGen.

### 3.5 Hospital course, diagnosis, and initial management during readmission

During readmission, the infant presented with persistent feeding difficulties and failure to thrive. Despite on-demand formula feeding and attempts at small, frequent feedings, his oral intake remained unstable, with frequent occurrences of sucking fatigue, feeding refusal, and postprandial vomiting. Growth parameter monitoring indicated that weight, length, and head circumference were persistently below the 3rd percentile for corrected age, consistent with postnatal growth retardation. Physical examination revealed hypotonia and decreased responsiveness, and neurological developmental assessment suggested potential delays in early developmental milestones.

To investigate the etiology of the feeding difficulties and optimize nutritional support, the following evaluations were performed:

1. Feeding Assessment and Gastrointestinal Function: Videofluoroscopic swallow study showed no definite structural swallowing disorder; upper gastrointestinal series suggested mild gastroesophageal reflux.
2. Metabolic and Endocrine Evaluation: Blood ammonia, lactate, glucose, thyroid function, and insulin-like growth factor-1 levels were all within normal limits.
3. Genetic Follow-up: Trio whole-exome sequencing results confirmed a hemizygous deletion of the SSR4 gene, which was highly consistent with the clinical presentation, leading to a diagnosis of SSR4-CDG (Congenital Disorder of Glycosylation type Iy).

Integrating the clinical presentation and investigative findings, the final diagnoses were: 1) Severe protein-energy malnutrition; 2) Multiple congenital heart defects (ventricular septal defect, atrial septal defect, persistent left superior vena cava) with cardiac insufficiency; and 3) SSR4-CDG.

A multidisciplinary, individualized management plan was initiated:

1. Cardiac Management: Due to signs of pulmonary congestion and cardiac insufficiency, treatment with deslanoside (for inotropic support) and furosemide (diuretic) was initiated. Surgical closure of the ventricular septal defect is planned within the first year of life.
2. Nutritional Support: Under the guidance of a feeding therapist, feeding was adjusted to thickened formula with gradual increases in caloric density. Due to inefficient oral intake, nasogastric tube feeding with high-calorie formula (24–27 kcal/oz) and hydrolyzed protein formula was initiated, resulting in a slow but positive weight gain trend.
3. Hematological Intervention: Given the presence of coagulopathy, vitamin K supplementation was administered, and preventive measures were taken for any invasive procedures.
4. Neurodevelopmental and Rehabilitation: Early physical therapy and occupational therapy interventions were started for hypotonia. Regular neurodevelopmental assessments were scheduled, and a referral was made to an early intervention program.
5. Genetic Counseling and Long-term Monitoring: Comprehensive genetic counseling was provided to the family. A plan was established for monitoring very long-chain fatty acids to screen for adrenoleukodystrophy (ALD). A long-term follow-up plan was developed in collaboration with the departments of Nutrition, Rehabilitation, Neurodevelopment, and Genetic Counseling.

After two weeks of this comprehensive management, the infant's feeding efficiency improved, and the frequency of vomiting decreased. Parental understanding of the disease and compliance with the management plan increased. The infant was discharged at 5 weeks of age on nasogastric tube feeds with coordinated outpatient multidisciplinary follow-up arranged. Collectively, these cardiac abnormalities resulted in clinically significant left-to-right shunting and early signs of heart failure, making cardiac disease an early and prominent manifestation in this patient.

## 4 Systematic literature review and detailed analysis

### 4.1 Literature search and study selection

A comprehensive search of PubMed, Embase, Google Scholar, CNKI, and Wanfang databases was conducted up to December 1, 2025, using the terms: “SSR4,” “SSR4-CDG,” “CDG1Y,” “congenital disorder of glycosylation type Iy,” “TRAP complex,” and associated variants. All English and Chinese peer-reviewed case reports and series were included. Data on demographics, genetics, clinical features, diagnostics, and outcomes were extracted. For analysis, patients were stratified into age groups: Neonatal/Infant (<1 years), Child (1–12 years), Adolescent/Adult (>12 years). Our patient was included, making a total cohort of ***n*** **=** **28** (Supplementary Figure S2).

### 4.2 Genotypic spectrum (*n* = 28)

Genetic profiling of the 28 reported SSR4-CDG cases (Table 1) reveals a heterogeneous mutational spectrum with diverse pathogenic variants distributed across the SSR4 gene. Variant types include frameshift mutations (e.g., c.316delT, p.F106Sfs53; c.358_359del, p.Arg120Glufs2), nonsense mutations (e.g., c.268C>T, p.Arg90; c.274C>T, p.Gln92), splice-site alterations (e.g., c.417+1G>A; c.442-1G>C), and partial or complete gene deletions. Notably, a subset of cases carries larger hemizygous deletions encompassing Xq28, which often include not only SSR4 but also adjacent genes such as SRPK3, IDH3G, PDZD4, PLXNB3, and ABCD1, suggesting possible genomic instability or non-allelic homologous recombination in this region.

**Table 1:** Comprehensive clinical and genetic profile of All reported SSR4-CDG patients (*n* = 28).

Patient ID (Source)	Age at report	Genetic variant (HGVS)	Inheritance	Core phenotype								
				Developmental delay	Intellectual disability	Muscular hypotonia	Abnormal facial features	Microcephaly	Feeding/GI	CHD	Epilepsy/EEG abnl.	cMRI abnl.
P1（Losfeld E,2014）	16y	c.316delT, p. F106Sfs*53	*de novo*	+	+	+	+	+	+	−	+	−
P2 (Ng et al., 2015)	10y	g.153062612_153063511del	*de novo*	+	+	+	+	+	+	−	+	−
P3 (Ng et al., 2015)	4y	c.358_359del, p.Arg120Glufs*2	Maternal mutation germline	+	+	+	+	+	+	−	−	−
P4 (Ng et al., 2015)	2y	c.358_359del, Arg120Glufs*2	Maternal mutation germline	+	+	+	+	+	+	−	−	−
P5 (Ng et al., 2015)	4y	g.153031975_153105401del	X-chromosomal (maternal)	+	+	+	+	+	+	−	+	+
P6 (Ng et al., 2015)	14y	c.417 1G>A	*de novo*	+	+	+	+	+	+	+	+	−
P7 (Ng et al., 2015)	13y	c.442-1G>C	X-chromosomal (maternal)	+	+	+	+	+	+	−	−	+
P8 (Ng et al., 2015)	5y	c.442-1G>C	X-chromosomal (maternal)	+	+	+	+	+	+	−	−	+
P9 (Ng et al., 2015)	ND	c.442-1G>C	X-chromosomal (maternal)	+	+	+	+	+	−	−	+	ND
P10(Medrano et al., 2019)	17y	c.180_183del, p. Phe60Leufs*6	*de novo*	+	+	+	+	+	−	−	+	+
P11(Ferreira et al, 2020)	11y	c.268C>T, p.Arg90*	*de novo*	+	+	+	+	+	+	+	−	+
P12(Castiglioni et al., 2021)-	12y	c.274C>T, p.Gln92*	*de novo*	+	+	+	+	+	+	−	+	+
P13(Castiglioni et al., 2021)	26m	arr [GRCh37]Xq28 (153011909_153063825) x0	*de novo*	+	+	+	+	+	+	−	−	+
P14(Castiglioni et al., 2021)	34m	arr [GRCh37]Xq28 (153060022_153063888) x0	Maternal	+	+	+	+	+	−	−	−	+
P15(Wang et al., 2022)	36m	c.269G>A, p.Trp90*	*de novo*	+	+	+	+	+	−	+	−	+
P16(Wu et al., 2022)	10y	c.302dupC, p. Y102Lfs*2	*de novo*	+	+	+	+	+	+	−	−	ND
P17(Weng et al., 2023)	4m	Deletion chrX: 153 045 645-153 095 809, including SRPK3, IDH3G, SSR4, PDZD4	*de novo*	+	ND	+	+	ND	+	ND	−	+
P18(Johnsen C, 2024)	17y	c.417+1G>A,	*de novo*	+	+	+	+	+	−	−	+	+
P19(Johnsen C, 2024)	5y	c141dup/p. Val48ArgfsTer22	*de novo*	+	+	+	+	+	+	−	−	−
P20(Johnsen C, 2024)	5y	Single copy number decrease of 64 kB on Xq28, containing SSR4, PLXNB3, SRPK3, IDH3G, SSR4, PDZD4 (partial)	*de novo*	+	+	+	+	+	+	+	+	+
P21(Johnsen C, 2024)	28y	c.302G>A/p. Trp101*	*de novo*	+	+	+	+	+	+	+	−	+
P22(Li, T.2024)	1.5y	g.153059779_153063888del	Maternal	+	+	+	+	+	+	−	+	−
P23(Li, T.2024)	8m	c.47_63del, p.Ser16Phefs*19	Maternal	+	+	+	+	+	+	+	−	−
P24(Lien JC,2025)	3m	single copy number decrease of 110kB on Xq28, containing BCAP31, ABCD1, PLXNB3, SRPK3, ICH3G, SSR4 and PDZD4	*de novo*	+	+	+	+	+	+	+	−	+
P25 (Sun W,2024)	8m	c.80_96del,p.Ser27Phefs*19	X-chromosomal (maternal)	+	+	+	+	+	+	+	−	ND
P26(Li N, 2025)	4y	c.351+1del	X-chromosomal (maternal)	+	+	ND	+	+	ND	−	+	−
P27(Wang Q,2025)	17y	c.67+2T>C	X-chromosomal (maternal)	+	+	+	+	+	+	−	+	−
P28 this study	1m	Single copy number decrease of 65.63kB on Xq28, containing ABCD1(partial), PLXNB3, SRPK3, ICH3G, SSR4 and PDZD4	X-chromosomal (maternal)	+	+	+	+	+	+	+	+	+

Feeding/GI = feeding difficulty or gastrointestinal involvement; cMRI: cranial magnetic resonance imaging; CHD=congenital heart disease;Presence or absence of clinical features is indicated as ‘+’ or ‘−’. Clinical terminology and phenotype categories are harmonized with Supplementary Tables S1, S2.

The table includes the following columns: Patient ID (Source), Age at Report, Genetic Variant (HGVS), Inheritance, and Core Phenotype. Core phenotypic manifestations encompass developmental delay, intellectual disability, muscular hypotonia, abnormal facial features, microcephaly, feeding difficulties or gastrointestinal involvement (Feeding/GI), congenital heart disease (CHD), epilepsy or abnormal EEG findings, and abnormal cranial magnetic resonance imaging (cMRI). Clinical features are consistently annotated as present (+) or absent (–) across all patients, with genetic inheritance patterns categorized as *de novo*, X-chromosomal (typically maternal), or maternal germline variants. This integrated overview highlights the uniform presence of developmental and neurological impairments in SSR4-CDG, alongside variable systemic involvement, and underscores the predominance of loss-of-function variants and X-linked transmission in this disorder.

Inheritance follows an X-linked recessive pattern, with approximately half of the cases (15/28, 53.6%) attributed to *de novo* mutations and the remainder (13/28, 46.4%) resulting from maternal transmission, consistent with germline or X-linked maternal inheritance. To date, no clear genotype–phenotype correlation has been established, as neither variant type (point mutation vs. deletion) nor location appears to reliably predict clinical severity or specific phenotypic features.

In summary, the genetic landscape of SSR4-CDG is characterized by loss-of-function variants in SSR4, an X-linked recessive inheritance with a high rate of *de novo* events, and the presence of multigenic deletions, which collectively complicate genetic counseling and prenatal diagnosis.

### 4.3 Phenotypic analysis: overall and age-stratified

Table 1 summarizes the complete cohort. The core clinical phenotype is highly consistent, predominantly characterized by: global developmental delay (28/28, 100%), intellectual disability (28/28, 100%), muscular hypotonia (28/28, 100%), characteristic facial dysmorphism (28/28, 100%), microcephaly (27/28, 96.4%), and feeding difficulties/gastrointestinal symptoms (23/28, 82.1%). Abnormal cranial magnetic resonance imaging findings were present in 53.6% (15/28), and epilepsy/electroencephalogram abnormalities were observed in 46.4% (13/28).

**Figure 4:**
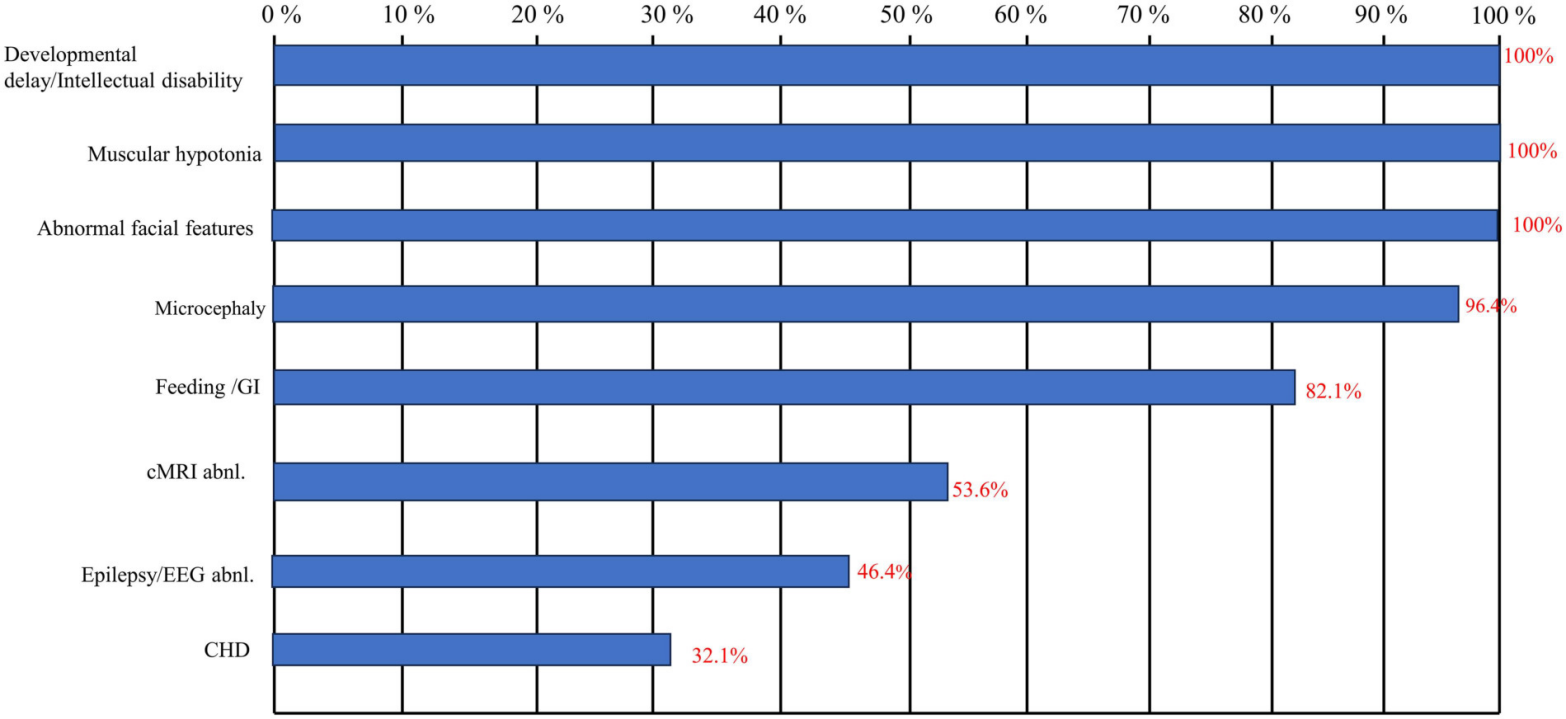
Frequency of systemic involvement in the SSR4-CDG cohort (*n* = 28). As shown in the figure, each bar represents the percentage of a core phenotype among individuals with SSR4-CDG, sorted by frequency.

Figure 4 illustrates the frequency of major systemic involvements across the entire cohort:

- **Global developmental delay/Intellectual disability:** 100% (28/28). This represents the most characteristic and universal manifestation of the disease.
- **Muscular hypotonia:** 100% (28/28). Present in the vast majority of patients and serves as a key clinical sign in infancy and early childhood.
- **Characteristic facial dysmorphism:** 100% (28/28). All patients exhibit distinctive facial features, providing important diagnostic clues.
- **Microcephaly:** 96.4% (27/28). Most patients present with head circumference below the normal range, often showing a progressive trend.
- **Feeding difficulties/Gastrointestinal symptoms:** 82.1% (23/28). Particularly prominent during infancy and early childhood, with partial improvement possible in older children, though symptoms often persist.
- **Abnormal cranial magnetic resonance imaging (cMRI):** 53.6% (15/28). Common findings include non-specific changes such as corpus callosum abnormalities, ventricular dilation, and white matter signal alterations.
- **Epilepsy/Electroencephalogram (EEG) abnormalities:** 46.4% (13/28). Presents in various forms, including focal seizures and febrile convulsions, with onset occurring across different ages.
- **Congenital heart defects (CHDs):** 32.1% (9/28). May include various types such as atrial septal defects, ventricular septal defects, and complex cardiac malformations.

Age-stratified Core Clinical Phenotypes, the patients were stratified into three groups: <1 year, 1–12 years, and >12 years. The core phenotypes were analyzed accordingly and are summarized as follows(See Supplementary Table S1):

- Infancy (<1 year, *n* = 5): The clinical presentation was dominated by feeding difficulties (4/5, 80%), muscular hypotonia (5/5, 100%), and characteristic facial dysmorphism (5/5, 100%). Congenital heart defects (CHDs) were identified in 2/5 (40%) of patients early in the course, offering an important diagnostic clue. Although neurodevelopmental delay was already evident, precise quantification remained challenging at this early stage.
- Childhood (1–12 years, *n* = 14): The hallmark diagnostic features became fully apparent, including global developmental delay (14/14, 100%), intellectual disability (13/14, 92.9%), and distinctive facial features (14/14, 100%). Microcephaly (13/14, 92.9%) was consistently present. Moreover, epilepsy or EEG abnormalities (7/14, 50%), cranial MRI abnormalities (9/14, 64.3%), and feeding/gastrointestinal involvement (13/14, 92.9%) were notably prominent in this age group.
- Adolescence and adulthood (>12 years, *n* = 9): The core phenotypes persisted, with a higher frequency of epilepsy or EEG abnormalities (7/9, 77.8%) and cranial MRI abnormalities (7/9, 77.8%). CHDs remained detectable in 4/9 (44.4%) of these older patients. Over time, behavioral or psychiatric disturbances, connective tissue involvement, and chronic gastrointestinal symptoms are likely to become more pronounced, significantly affecting long-term quality of life and posing ongoing management challenges.

In-depth analysis of cardiac involvement: Although CHDs are present in more than one-quarter of all patients, the present case is notable for two distinctive aspects: (1) severity and complexity–a hemodynamically significant ventricular septal defect (VSD) combined with an atrial septal defect (ASD) and vascular anomalies represents the most complex cardiac phenotype reported to date; and (2) mode of presentation–in all previously reported cases, CHDs were incidentally identified during evaluation for developmental delay. In contrast, in our patient, CHDs were the primary reason for neonatal intensive care and served as a catalyst for prompt genetic diagnosis.

In summary, the phenotype of SSR4-CDG demonstrates clear age-dependent evolution: infants present primarily with feeding, tone, and early structural abnormalities; children exhibit fully expressed neurodevelopmental and facial features along with emerging neurological and systemic involvement; and adolescents/adults show consolidation of neurological abnormalities and increasing systemic complications. These observations reinforce the need for age-specific, multidisciplinary follow-up throughout the lifespan.

### 4.4 Patient and public involvement

No patients or members of the public were involved in the design, conduct, or reporting of this study due to its retrospective and genetic diagnostic nature.

## 5 Discussion

This case expands the phenotypic spectrum of SSR4-CDG to include severe neonatal structural heart malformations as a potential early presentation. Beyond phenotypic expansion, this report contributes to the field in three principal ways: 1. It documents the earliest postnatal molecular diagnosis reported to date. 2. It provides detailed cardiovascular phenotyping in the neonatal period. 3. It offers an updated age-stratified synthesis of all published cases, highlighting developmental evolution of clinical manifestations.

### 5.1 Potential mechanistic link between SSR4 deficiency and cardiac development

The severe CHDs observed in this patient may reflect impaired N-glycosylation during early cardiogenesis. Cardiac septation and vascular patterning depend on tightly regulated signaling pathways, including Notch and FGF signaling, both of which involve heavily glycosylated receptors and ligands (23–26).

Disruption of the TRAP complex due to SSR4 loss may impair proper folding and trafficking of these signaling molecules during a critical developmental window (weeks 3–8 of gestation), potentially contributing to septal defects such as VSD and ASD. Although this mechanistic link remains hypothetical and requires functional validation, the recurrence of structural cardiac defects in a subset of reported SSR4-CDG cases supports a biologically plausible association.

Among previously reported cases, Patient 21 described in the 2024 review by Johnsen et al. (6, 27) also exhibited cardiovascular abnormalities. However, in that case, cardiac findings were not reported as the primary clinical concern during the neonatal period, nor were they described as hemodynamically significant at presentation. In contrast, our patient required early cardiologic management due to clinically relevant left-to-right shunting and signs of heart failure. This comparison suggests variability in cardiac severity rather than increased prevalence.

### 5.2 Comparison with cardiac involvement in other CDG types

Cardiac manifestations are well-documented in several CDG types, most notably **PMM2-CDG (CDG-Ia)**, the most common N-linked CDG. In PMM2-CDG, pericardial effusions and cardiomyopathy are frequent, often presenting in infancy and contributing to high mortality (28). Structural heart defects like ASD and VSD also occur but are less common than functional myocardial disease. The pathophysiology differs: PMM2 deficiency disrupts the synthesis of the LLO precursor, causing a more severe, global glycosylation defect from the earliest steps. This leads to widespread organ dysfunction, including severe liver disease and endocrine deficiencies, alongside cardiac issues.

In contrast, **SSR4-CDG** appears to cause a somewhat “milder” overall glycosylation defect, as suggested by survival into adulthood and less severe visceral involvement. However, our case demonstrates that its impact on specific developmental processes, like cardiac septation, can be profound. This highlights **phenotypic specificity** within CDGs; defects in different steps of the glycosylation pathway (e.g., LLO synthesis vs. protein translocation/glycosylation complex) can preferentially affect different tissues or developmental programs. Unlike PMM2-CDG where cardiomyopathy dominates, SSR4-CDG may be more strongly associated with **structural congenital heart defects**, a distinction important for anticipatory care.

### 5.3 Phenotypic attribution in the context of a contiguous gene deletion

It is important to interpret the clinical phenotype in light of the contiguous Xq28 deletion identified in this patient. While complete loss of SSR4 is sufficient to cause SSR4-CDG, the concurrent deletion of adjacent genes—including ABCD1, SRPK3, IDH3G, PLXNB3, and PDZD4—may potentially modulate certain aspects of the phenotype.

Core features such as global developmental delay, hypotonia, facial dysmorphism, microcephaly, feeding difficulties, and coagulation abnormalities are highly consistent across previously reported SSR4-CDG cases without contiguous deletions. Therefore, these manifestations are most plausibly attributable to SSR4 deficiency.

However:

- ABCD1 deletion introduces a risk for X-linked adrenoleukodystrophy and may contribute to long-term neurological vulnerability (29).
- SRPK3 deletion has been associated with myopathic phenotypes and may theoretically exacerbate hypotonia.
- The contribution of IDH3G, PLXNB3, and PDZD4 deletions remains less clearly defined but cannot be fully excluded.

Importantly, severe congenital heart defects have been reported in a subset of SSR4-CDG patients without ABCD1 involvement, suggesting that cardiac anomalies are not solely attributable to the contiguous deletion (6).

Nevertheless, given the multigenic deletion in this case, phenotypic attribution should be interpreted with caution, and over-assignment to a single gene should be avoided.

### 5.4 Complex phenotype and comprehensive management of a contiguous gene deletion syndrome

This case involves a contiguous deletion at Xq28 encompassing SSR4 and five adjacent genes (ABCD1, SRPK3, IDH3G, PLXNB3, and PDZD4). The clinical phenotype likely results from the combined effect of the core congenital disorder of glycosylation due to complete SSR4 loss and the superimposed impact of the co-deleted genes. Notably, the partial deletion of ABCD1 introduces a lifelong risk for X-linked adrenoleukodystrophy, necessitating a systematic surveillance protocol including regular monitoring of very-long-chain fatty acids, adrenal function, and brain MRI. The deletion of SRPK3 may be associated with significant muscular hypotonia, warranting intensive neurorehabilitation. The deletions of IDH3G, PLXNB3, and PDZD4 could potentially affect metabolic homeostasis and synergistically exacerbate neurodevelopmental impairment, respectively. Consequently, the management of patients with such a contiguous gene deletion syndrome must be proactive, multidisciplinary, and systematic, encompassing neurodevelopmental, endocrine-metabolic, neuromuscular, and potential skeletal systems.

### 5.5 Expanded multidisciplinary management guidelines for SSR4-CDG

Based on our review and this complex case, we propose an enhanced management framework (A detailed age-stratified multidisciplinary management framework is summarized in Supplementary Table S2):

- **Neonatal Period:** Focus on stabilization: **Cardiology** evaluation (echo), **Hematology** workup (coagulation factors), **Nutrition** (high-calorie, often tube feeding), and **Neurology** (baseline MRI, EEG if seizures). **Genetics** confirms diagnosis and coordinates family testing.
- **Infancy/Childhood: Developmental Pediatrics** coordinates early intervention (PT, OT, Speech). **Ophthalmology** and **Orthopedics** evaluations begin. **Gastroenterology** manages reflux/constipation. **Endocrinology** monitors growth; consider GH therapy if indicated. **Behavioral Pediatrics/Psychiatry** assesses for emerging issues.
- **Adolescence/Adulthood: Mental health support** becomes paramount. **Orthopedics** manages progressive scoliosis/arthritis. **Cardiology** monitors for late-onset cardiomyopathy. **Endocrinology** assesses puberty and bone health. **Transition planning** to adult care services is essential.
- **For cases with *ABCD1* involvement:** Integrate **Metabolic Neurology** for ALD surveillance (annual VLCFAs, cortisol, brain MRI) and **Endocrinology** for adrenal monitoring.

#### 5.5.1 Therapeutic outlook

Currently, no disease-modifying therapy exists for SSR4-CDG. Management remains supportive and multidisciplinary, focusing on cardiac care, nutritional optimization, neurodevelopmental support, and surveillance for potential comorbidities associated with contiguous gene deletions. Future research into targeted molecular correction or modulation of endoplasmic reticulum-associated pathways may provide potential therapeutic directions.

### 5.6 Limitations

Several limitations should be considered in this study. First, the single-case design inherently restricts the generalizability of the findings; further validation through multicentre cohort studies is warranted. Second, the absence of functional assays leaves the mechanistic link between the genetic deletion and impaired TRAP-complex assembly/glycosylation unverified. Finally, the relatively short follow-up period precludes definitive conclusions regarding long-term neurodevelopmental trajectory, cardiac outcomes, and potential phenotypic contributions of co-deleted adjacent genes.

## 6 Conclusion

This report delineates a severe neonatal presentation of SSR4-CDG, broadening its phenotype to include life-threatening congenital heart defects as an early and clinically significant manifestation.We report the second largest *SSR4* deletion and the earliest postnatal diagnosis, facilitated by prenatal genetic screening. The contiguous deletion involving *ABCD1* adds a second serious diagnostic consideration (X-ALD) requiring distinct, lifelong surveillance. Our comprehensive literature review consolidates the genotypic and phenotypic landscape of this ultra-rare disorder, revealing age-dependent expression patterns. This case underscores critical clinical lessons: 1) SSR4-CDG should be considered in male neonates with syndromic CHDs, especially with suggestive facies or coagulopathy; 2) Prenatal CNV findings demand prompt postnatal phenotyping; 3) Management is lifelong, multidisciplinary, and must now account for potential comorbid conditions like ALD. Increased recognition of this expanded spectrum will improve diagnostic rates, family counseling, and patient care.

## Data Availability

The datasets presented in this study can be found in online repositories. The names of the repository/repositories and accession number(s) can be found in the article/Supplementary Material.

## Supplementary material

Supplementary Figure S1
Cardiac CTA findings in a neonatal patient with SSR4-CDG.
**S1(A, B)** Coronal reconstructions of the **(A)** right and **(B)** left pulmonary arteries. **S1(C, D)** Measurement sites at the proximal and distal bifurcation of the **(C)** right and **(D)** left pulmonary arteries. *CTA, computed tomography angiography.*

Supplementary Figure S2
PRISMA 2020 flow diagram illustrating the literature identification, screening, eligibility, and inclusion process for the systematic review of SSR4-CDG cases.
Flow diagram illustrating the identification, screening, eligibility assessment, and inclusion of studies for the systematic review. A total of 29 records were identified, from which 13 studies met the inclusion criteria for final analysis. (Adapted from the PRISMA 2020 statement.).

## References

[1] Jaeken J Péanne R. What is new in CDG? J Inherit Metab Dis. (2017) 40(4):569–86. 10.1007/s10545-017-0050-6 28484880

[2] Francisco R Brasil S Poejo J Jaeken J Pascoal C Videira PA Congenital disorders of glycosylation (CDG): state of the art in 2022. Orphanet J Rare Dis. (2023) 18(1):329. 10.1186/s13023-023-02879-z 37858231 PMC10585812

[3] Ng BG Freeze HH Himmelreich N Blau N Ferreira CR. Clinical and biochemical footprints of congenital disorders of glycosylation: proposed nosology. Mol Genet Metab. (2024) 142(1):108476. 10.1016/j.ymgme.2024.108476 38653092 PMC11251693

[4] Freeze HH Chong JX Bamshad MJ Ng BG. Solving glycosylation disorders: fundamental approaches reveal complicated pathways. Am J Hum Genet. (2014) 94(2):161–75. 10.1016/j.ajhg.2013.10.024 24507773 PMC3928651

[5] Ng BG Freeze HH. Perspectives on glycosylation and its congenital disorders. Trends Genet. (2018) 34(6):466–76. 10.1016/j.tig.2018.03.002 29606283 PMC5959770

[6] Johnsen C Tabatadze N Radenkovic S Botzo G Kuschel B Melikishvili G SSR4-CDG, an ultra-rare X-linked congenital disorder of glycosylation affecting the TRAP complex: review of 22 affected individuals including the first adult patient. Mol Genet Metab. (2024) 142(3):108477. 10.1016/j.ymgme.2024.108477 38805916

[7] Losfeld ME Ng BG Kircher M Buckingham KJ Turner EH Eroshkin A A new congenital disorder of glycosylation caused by a mutation in SSR4, the signal sequence receptor 4 protein of the TRAP complex. Hum Mol Genet. (2014) 23(6):1602–5. 10.1093/hmg/ddt550 24218363 PMC3929095

[8] Tingting L Xiaolong D Songwei G Chunming R Yanping L Liu Y X chromosome microdeletion involving the SSR4 gene leading to congenital disorder of glycosylation: a case report and literature review. Chin J Pediatr Emerg Med. (2024) 31(12):958–61. 10.3760/cma.j.issn.1673-4912.2024.12.016

[9] Li N Chen C. Novel SSR4 gene splice variant leads to congenital disorder of glycosylation, type iy. Front Pediatr. (2025) 13:1651524. 10.3389/fped.2025.1651524 41210240 PMC12592166

[10] Wang Q Wang G Liang B Zhang C Yan C Lin P Intron retention caused by a canonical splicing variant in SSR4-related congenital disorder of glycosylation. J Hum Genet. (2025) 70(3):171–6. 10.1038/s10038-024-01309-7 39653760

[11] Lien JC Chandrasekaran VD Narumanchi TM Napoli MS Peterson JEG Williams CA. Hemizygous contiguous gene deletion within Xq28 that includes BCAP31, ABCD1, SRPK3 and SSR4: case report and literature review. Glob Med Genet. (2025) 12(3):100066. 10.1016/j.gmg.2025.100066 40662097 PMC12256310

[12] Sun W Jin X Zhu X. A novel SSR4 variant associated with congenital disorder of glycosylation: a case report and related analysis. Front Genet. (2024) 15:1402883. 10.3389/fgene.2024.1402883 39086474 PMC11288868

[13] Ng BG Raymond K Kircher M Buckingham KJ Wood T Shendure J Expanding the molecular and clinical phenotype of SSR4-CDG. Hum Mutat. (2015) 36(11):1048–51. 10.1002/humu.22856 26264460 PMC4604052

[14] Castiglioni C Feillet F Barnerias C Wiedemann A Muchart J Cortes F Expanding the phenotype of X-linked SSR4-CDG: connective tissue implications. Hum Mutat. (2021) 42(2):142–9. 10.1002/humu.24151 33300232

[15] Wang J Gou X Wang X Zhang J Zhao N Wang X. Case report: the novel hemizygous mutation in the SSR4 gene caused congenital disorder of glycosylation type iy: a case study and literature review. Front Genet. (2022) 13:955732. 10.3389/fgene.2022.955732 36386804 PMC9643473

[16] Shrimal S Cherepanova NA Gilmore R. Cotranslational and posttranslocational N-glycosylation of proteins in the endoplasmic reticulum. Semin Cell Dev Biol. (2015) 41:71–8. 10.1016/j.semcdb.2014.11.005 25460543 PMC4442082

[17] Pfeffer S Dudek J Schaffer M Ng BG Albert S Plitzko JM Dissecting the molecular organization of the translocon-associated protein complex. Nat Commun. (2017) 8:14516. 10.1038/ncomms14516 28218252 PMC5321747

[18] Hartmann E Görlich D Kostka S Otto A Kraft R Knespel S A tetrameric complex of membrane proteins in the endoplasmic reticulum. Eur J Biochem. (1993) 214(2):375–81. 10.1111/j.1432-1033.1993.tb17933.x 7916687

[19] Braunger K Pfeffer S Shrimal S Gilmore R Berninghausen O Mandon EC Structural basis for coupling protein transport and N-glycosylation at the mammalian endoplasmic reticulum. Science. (2018) 360(6385):215–9. 10.1126/science.aar7899 29519914 PMC6319373

[20] Russo A. Understanding the mammalian TRAP complex function(s). Open Biol. (2020) 10(5):190244. 10.1098/rsob.190244 32453970 PMC7276530

[21] Ng BG Lourenço CM Losfeld ME Buckingham KJ Kircher M Nickerson DA Mutations in the translocon-associated protein complex subunit SSR3 cause a novel congenital disorder of glycosylation. J Inherit Metab Dis. (2019) 42(5):993–7. 10.1002/jimd.12091 30945312 PMC6739144

[22] Freeze HH Ng BG. Golgi Glycosylation and human inherited diseases. Cold Spring Harb Perspect Biol. (2011) 3(9):a005371. 10.1101/cshperspect.a005371 21709180 PMC3181031

[23] Bruneau BG. The developmental genetics of congenital heart disease. Nature. (2008) 451(7181):943–8. 10.1038/nature06801 18288184

[24] Stanley P. Golgi glycosylation. Cold Spring Harb Perspect Biol. (2011) 3(4):a005199. 10.1101/cshperspect.a005199 21441588 PMC3062213

[25] Varshney S Stanley P. Multiple roles for O-glycans in notch signalling. FEBS Lett. (2018) 592(23):3819–34. 10.1002/1873-3468.13251 30207383 PMC6289669

[26] High FA Epstein JA. The multifaceted role of notch in cardiac development and disease. Nat Rev Genet. (2008) 9(1):49–61. 10.1038/nrg2279 18071321

[27] Verde A Cutri' MR Pagani F Pilotta A Pinelli L Asaro A Letter to the editors: concerning “SSR4-CDG, an ultra-rare X-linked congenital disorder of glycosylation affecting the TRAP complex: review of 22 affected individuals including the first adult patient” by Johnsen et al. Mol Genet Metab. (2025) 145(2):109136. 10.1016/j.ymgme.2025.109136 40367686

[28] Altassan R Péanne R Jaeken J Barone R Bidet M Borgel D International clinical guidelines for the management of phosphomannomutase 2-congenital disorders of glycosylation: diagnosis, treatment and follow up. J Inherit Metab Dis. (2019) 42(1):5–28. 10.1002/jimd.12024 30740725

[29] Mallack EJ Gao K Engelen M Kemp S. Structure and function of the ABCD1 variant database: 20 years, 940 pathogenic variants, and 3400 cases of adrenoleukodystrophy. Cells. (2022) 11(2):283. 10.3390/cells11020283 35053399 PMC8773697

