# Supplementary material for: Early neonatal diagnosis of SSR4-related congenital disorder of glycosylation with severe congenital heart defects: a case report and systematic review
Source: Front Pediatr. 2026 Mar 25;14:1780997. doi: 10.3389/fped.2026.1780997 (PMC13057274; doi:10.3389/fped.2026.1780997)
Supplement: Supplementary Figure S1 — Cardiac CTA findings in a neonatal patient with SSR4-CDG. S1(A, B) Coronal reconstructions of the (A) right and (B) left pulmonary arteries. S1(C, D) Measurement sites at the proximal and distal bifurcation of the (C) right and (D) left pulmonary arteries. CTA, computed tomography angiography. [file Table1.docx]

**Supplementary Table S1. Age-Stratified Frequency of Major Clinical Features in SSR4-CDG**

| Clinical feature | Neonatal / Infant onset (＜1 year) n (%) | Childhood onset (1-12 year) n (%) | Adolescence and adulthood (>12 years) n (%) |
| --- | --- | --- | --- |
| Developmental delay | 5/5 (100%) | 14/14 (100%) | 9/9 (100%) |
| Intellectual disability | 5/5 (100%) | 14/14 (100%) | 9/9 (100%) |
| Muscular hypotonia | 5/5 (100%) | 14/14 (100%) | 9/9 (100%) |
| Abnormal facial features | 5/5 (100%) | 14/14 (100%) | 9/9 (100%) |
| Microcephaly | 4/5 (80%) | 13/14 (92.9%) | 8/9 (88.9%) |
| Feeding /GI | 4/5 (80%) | 13/14 (92.9%) | 7/9 (77.8%) |
| Congenital heart disease (CHD) | 2/5 (40%) | 5/14 (35.7%) | 4/9 (44.4%) |
| Epilepsy/EEG abnl. | 1/5 (20%) | 7/14 (50%) | 7/9 (77.8%) |
| cMRI abnl. | 3/5 (60%) | 9/14 (64.3%) | 7/9 (77.8%) |
| Inheritance Pattern^*^(n/n) | *de novo*: 2/5 (40%) *X-chromosomal (maternal)*: 3/5 (60%) | *de novo*: 8/14 (57.1%) *X-chromosomal (maternal)*: 6/14 (42.9%) | *de novo*: 5/9 (55.6%) *X-chromosomal (maternal)*: 4/9 (44.4%) |

Supplementary Table S1 notes

Age stratification is based on age at clinical presentation. Percentages are calculated using the total number of patients in each age group, *indicates the proportion of de novo/X‑chromosomal (maternal) inheritance. Clinical feature categories and terminology are consistent with Table 1 and Supplementary Table S2.
