# Supplementary material for: Early neonatal diagnosis of SSR4-related congenital disorder of glycosylation with severe congenital heart defects: a case report and systematic review
Source: Front Pediatr. 2026 Mar 25;14:1780997. doi: 10.3389/fped.2026.1780997 (PMC13057274; doi:10.3389/fped.2026.1780997)
Supplement: Supplementary Figure S2 — PRISMA 2020 flow diagram illustrating the literature identification, screening, eligibility, and inclusion process for the systematic review of SSR4-CDG cases. Flow diagram illustrating the identification, screening, eligibility assessment, and inclusion of studies for the systematic review. A total of 29 records were identified, from which 13 studies met the inclusion criteria for final analysis. (Adapted from the PRISMA 2020 statement.). [file Table2.docx]

**Supplementary Table S2. Multidisciplinary Management Recommendations for SSR4-CDG**

| **Management Across the Lifespan (Bullet-Style)** | |
| --- | --- |
| **Life stage** | **Key management points** |
| **Neonatal / Infant period(＜1 year)**   \| • Baseline evaluation: neurologic exam ± EEG, brain MRI if indicated, echocardiography (CHD), ophthalmologic exam \| \| --- \|  \| • Feeding assessment and nutritional support; manage GERD and failure to thrive; consider tube feeding \| \| --- \|  \| • Coagulation testing if bleeding tendency suspected \| \| --- \|  \| • Early initiation of PT/OT and feeding therapy \| \| --- \|  \| • Parental testing and X-linked genetic counseling \| \| --- \| | **•** Molecular confirmation by WES and/or CNV analysis in neonates with hypotonia, feeding difficulty, and multisystem involvement |
| **Childhood(1-12 year)**   \| • Seizure surveillance and treatment; repeat EEG as clinically indicated \| \| --- \|  \| • Continued PT/OT/speech therapy \| \| --- \|  \| • System-based follow-up: GI/nutrition, orthopedics (joint laxity, scoliosis), ophthalmology \| \| --- \|  \| • Re-evaluation of coagulation status before surgical procedures \| \| --- \| | **•** Regular monitoring of growth, neurodevelopment, and behavior (including ASD-like features) |
| **Adolescence and adulthood**(>12 years)   \| • Follow-up of long-term neurologic, cognitive, and functional outcomes \| \| --- \|  \| • Psychosocial and mental health support \| \| --- \|  \| • Adult subspecialty follow-up (cardiology, endocrinology, rehabilitation) as indicated \| \| --- \| | **•** Planned transition to adult multidisciplinary care |
| **Special consideration: *ABCD1* contiguous deletion**   \| • Periodic screening for adrenal insufficiency (ACTH, cortisol), even if asymptomatic \| \| --- \|  \| • Long-term neurologic surveillance for X-ALD–related manifestations \| \| --- \|  \| • Targeted family counseling addressing combined SSR4-CDG and X-ALD risks \| \| --- \| | **•** Early differentiation from X-linked adrenoleukodystrophy (X-ALD), especially in males |

**Table notes**
CHD = congenital heart disease; ASD = autism spectrum disorder; PT/OT = physical/occupational therapy; WES = whole-exome sequencing; CNV = copy number variation. Clinical terminology and age stratification are harmonized with Table 1 and Supplementary Table S1.
