# Supplementary figures and images for: Early neonatal diagnosis of SSR4-related congenital disorder of glycosylation with severe congenital heart defects: a case report and systematic review
Source: Front Pediatr. 2026 Mar 25;14:1780997. doi: 10.3389/fped.2026.1780997 (PMC13057274; doi:10.3389/fped.2026.1780997)

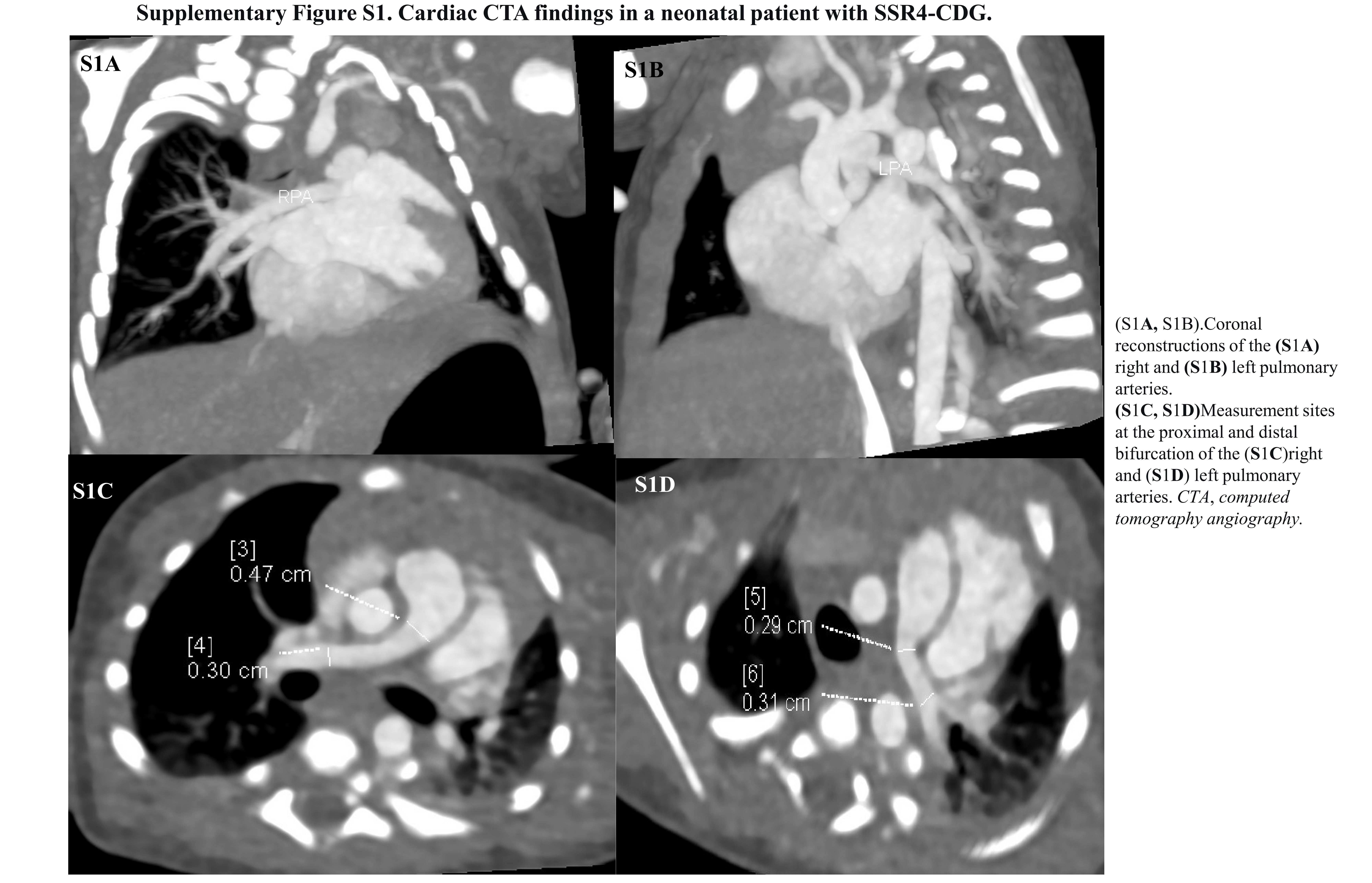

Supplement: Supplementary file 3 [file Image1.jpeg]

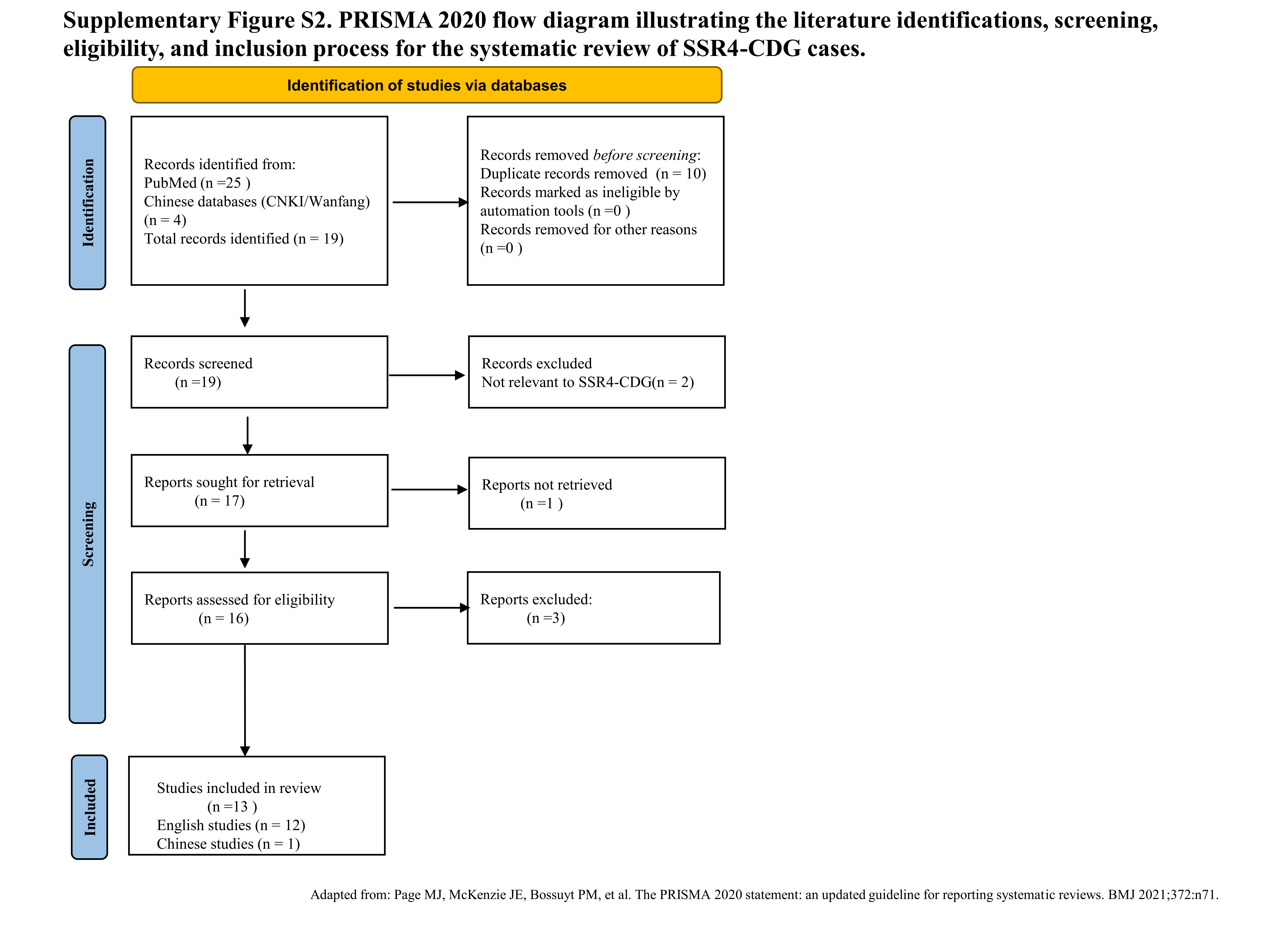

Supplement: Supplementary file 4 [file Image2.jpeg]
